# Health Related Quality of Life among Omani Men and Women with Type 2 Diabetes

**DOI:** 10.1155/2016/8293579

**Published:** 2015-11-22

**Authors:** Melba Sheila D'Souza, Ramesh Venkatesaperumal, Susan D. Ruppert, Subrahmanya Nairy Karkada, Devakirubai Jacob

**Affiliations:** ^1^Department of Adult Health and Critical Care, College of Nursing, Sultan Qaboos University, 123 Muscat, Oman; ^2^The University of Texas Health Science Center at Houston School of Nursing, Houston, TX 77030, USA; ^3^Department of Business Studies, Higher College of Technology, 123 Al-Khuwair, Oman

## Abstract

The aim of this study was to explore predictors of health related quality of life (HRQoL) among men and women with type 2 diabetes. This cross-sectional descriptive study consisted of a random sample of 300 adults with type 2 diabetes in a selected public hospital. Euro-QoL and Revised Summary of Diabetes Self-Care Activities scales were used to collect data between January and June 2010. Schooling and ability to manage positively were highly significant predictors of quality of life (QoL) among women as compared to men. Age, prevention of activities of daily living and knowledge/management of diabetes were significant predictors of Health state among women as compared to men. Findings demonstrate that 30.6% (versus 35.7%) of the variance in the total QoL and 14% (versus 23%) of the variance in health state could be explained by personal and clinical characteristics among women and men, respectively. The study underlines the importance for nurse educators to assess HRQoL among men and women and to develop effective self-care management strategies based on personal and clinical characteristics.

## 1. Introduction

Diabetes mellitus (DM) is a chronic progressive metabolic disorder due to absolute (type 1) or relative (type 2) deficiency of insulin hormone [1]. Worldwide, 366 million people were estimated to have diabetes mellitus in the year 2011, and numbers are predicted to double by 2030 [2–4]. DM has caused approximately 4.6 million deaths in the age group of 20–79 years in a ten-year period from 2001 to 2011, accounting for approximately 8.2% of mortality [5, 6]. Almost 80% of deaths related to diabetes occur in low- and middle-income developing countries [7]. The incidence of type 2 diabetes (T2D) with an early onset associated with complications has risen in recent years in Oman compared to other Middle East countries [8–10]. The impact of T2D may limit function and quality of life among men and women. Individuals with T2D need a disciplined balance between the demands of self-care and preferred lifestyles.

Type 2 diabetes is developing into an international public health problem, with a significant increase in the Middle East region [6, 11]. In Oman, the prevalence of T2D escalated from 11.6% (2000) to 15% (2005) and rose to 16.1% (2010), with rising prevalence among all age groups [12, 13]. These figures are expected to double by 2030 [11] due to the life threatening long term complications [14, 15] and substantial impact on health and well-being [16–18]. A significant number of Omani men and women lack knowledge, skills, and information on self-care management while coping with T2D [8]. Hence, an exploration of personal and clinical factors to improve self-care behaviors among Omani men and women with T2D is important in assisting them in managing their health.

## 2. Review of Literature

**Figure 1:**
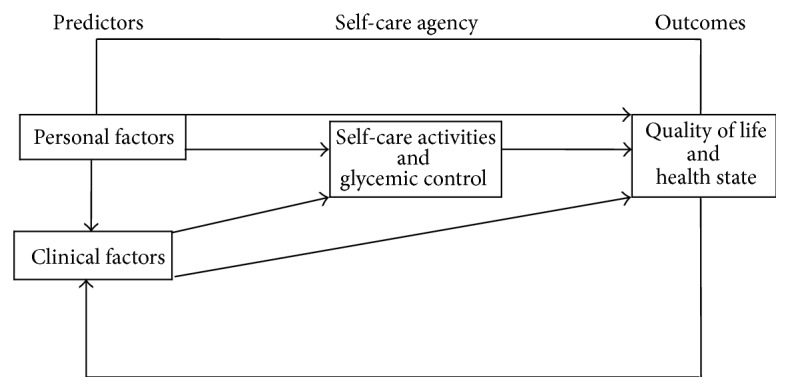
Health related quality of life among adults with type 2 diabetes (adapted from Sousa et al. [33]).

Health related quality of life (HRQoL) is a multidimensional construct with bearing on a person's physical, cognitive, social, emotional, psychological, role, and spiritual status [19, 20]. HRQOL is an acceptable outcome or efficacy of self-care among adults with T2D (Figure 1). The literature on perceptions of living with T2D is extensive and has been shown to correlate with quality of life (QoL) [21, 22]. Several studies show that adults with T2D rate their QoL lower than the general population [23–26] as compared to those with type 1 diabetes (T1D) [27, 28]. Women with T2D have been found to have a lower quality of life than men [29–31], and those with a longer duration of T2D had poor QoL [32]. A self-care management model [33] leads to better glycemic control [34] and QoL [35], while those with poor glycemic control were found to have low QoL [36, 37]. No studies focusing on the predictors of QoL and health status among Omani men and women have been reported. Hence, the purpose of this study was to examine the predictors of QoL and health state and to examine comparisons among Omani men and women.

## 3. Material and Methods

### 3.1. Design

A cross-sectional descriptive study was conducted among adults with T2D in the outpatient diabetes clinic in a selected public hospital.

### 3.2. Sample/Participants

A sampling framework list of the accessible population with known type 2 diabetes (*N* = 2000) was obtained from the diabetes clinic in a selected public tertiary hospital in Oman. A simple random sampling using random number tables was used to recruit Omani adults who were screened in this diabetes clinic. The inclusion criteria were adults above 18 years diagnosed with T2D for two years who were able to understand, communicate, and converse in Arabic or English language and were not currently pregnant. The exclusion criteria were adults with known diagnosis of T1D, unknown T2D, cognitive/neurological impairment, mental/physical disability, or critical or advanced complications.

Sample size was estimated with the G^*∗*^Power software at a power of 0.95 with an effect size of 0.15 using 10 predictors (independent variables), an alpha of 0.05, and standard deviation of 1% on two-tailed testing [38, 39]. To obtain a power of 0.95 and assuming a 30% incompletion rate, a total sample size of 330 was required for these input parameters. Subsequently, a random sample of 330 adults with T2D was recruited.

### 3.3. Measurement

A review of the literature was conducted to select standardized instruments to measure the identified concepts of HRQoL and health/self-care activities. Two instruments were reviewed and selected. Physiological indicators were used to assess diabetes control and body weight. The sociodemographic and clinical-related information was primarily gathered by a demographic and clinical baseline tool which included age, gender, schooling (educational level), duration of diabetes, diabetes education, knowledge and management, ability to manage, activities of daily living, and medication.

Health related quality of life was evaluated with the EuroQol (EQ-5D) [40, 41]. The EQ-5D-5L consists of five dimensions (mobility, self-care, usual activities, pain/discomfort, and anxiety/depression). Each dimension had five levels: no problems, slight problems, moderate problems, severe problems, and extreme problems. The EQ visual analog scale (VAS) then recorded the respondent's self-rated health on a 20 centimeter (10-point interval) vertical VAS with endpoints categorized as “the best health you can imagine” and “the worst health you can imagine.” Worst imaginable health state was recorded as 0 at the bottom of the scale, and best imaginable health state was achieved as 100 at the top. Both the 5-item index score and the VAS score were then converted into a value score between 0 (“worst health state”) and 1 (“best health state”) [24, 40].

Self-care activities (SCA) were evaluated with the Revised Summary of Diabetes Self-Care Activities Scale (SDSCA) to assess aspects of the diabetes regimen and evaluate the dietary management skills of the participants. The SDSCA scale is a self-reporting measure of the frequency of performing 13 diabetes self-care tasks and consisted of six subscales of the diabetes self-management (DSM) behaviors: diet, exercise, blood glucose testing, medication taking, foot care, and smoking behavior over the prior seven days [42]. Interitem correlations had a range of *r* = 0.20–0.76 (mean = 0.47) for four SDSCA subscales and 6-month test-retest reliability had a range of *r* = 0.00–0.58 (mean = 0.40) [42].

The glycosylated or glycated hemoglobin (HbA1c) value was classified into good glycemic control if the HbA1c values are less than or equal to (≤) 7% and poor glycemic control if HbA1c values are greater than (>) 7%. Glycemic control of ≤7.0% is endorsed as a treatment goal [17, 43]. Body mass index (BMI) in weight in kilograms/square of height in meters (Kg/m^2^) was categorized as underweight if ≤18.5 Kg/m^2^, normal if 18.5–24.9 Kg/m^2^, overweight if 25–29.9 Kg/m^2^, and obese if ≥30 Kg/m^2^ [44]. Weight and height were measured by a portable digital scale and a portable stadiometer.

### 3.4. Validity and Reliability

The EQ-5D, SDSCA, and demographic and clinical characteristics instruments were translated to Arabic and back-translated and checked with monolingual testing. No discrepancies were found between the original and linguistic translated versions of the instruments. The linguistic validation of the Arabic version of the tools was found to be adequate. The final instruments used in this study were administered to 30 Omani adults with T2D twice in a 2-week interval. Intraclass correlation coefficient was intended to evaluate the test-retest reliability for the subdimensions of the EQ-5D (0.75 and 0.91). Interitem correlations had a range of *r* = 0.75–0.86 for four SDSCA subscales and item-to-total correlations had a range of 0.77–0.91 for the SDSCA. Good evidence for internal consistency was shown using Cronbach's alpha for the SDSCA which demonstrated *α* = 0.87, which was considered acceptable.

### 3.5. Data Collection

Data were collected using EQ-5D and SDSCA standardized questionnaires after the pilot study between January and June 2010 among 330 adults with T2D in the diabetes clinic. Study participants were provided with an explanation of the study, and informed consent was obtained. Ethical approval was provided by the Ethics and Research Committee, College of Nursing at Sultan Qaboos University. Informed written and verbal consent was acquired from each participant who met the inclusion criteria through a written letter. Confidentiality was retained between the investigator and the participant. Informed consent and the completed questionnaires were stored and kept in locked cabinets. During the study, 30 selected adults dropped from the study. A 91% (*N* = 300) completion response rate was obtained.

## 4. Data Analysis and Results

Study surveys and biological samples were labeled with a unique study identifier. Coded data files were kept separately from the code list to maintain anonymity. The Statistical Packages for Social Sciences (SPSS) was used for analysis. A probability of <0.05 was considered statistically significant. Descriptive and inferential statistics were calculated using the SPSS statistical package version 21.0 (SPSS Inc., Chicago, IL, USA). A normality test and multicollinearity checks were performed. The determinants of QoL were assessed with ANOVA and multivariate generalized linear model (GLM)/MANOVA [45–48]. Predictors were determined for MANOVA using important determinants from ANOVA [47].

### 4.1. Demographic and Clinical Characteristics (Table 1)

The highest percent of Omani men was between 50–59 years (40.6%) as compared to 21.7% among the women (Table 1). The highest frequency of adherence to self-care activities was an average of three days/week among men (61.5%) and women (60.5%). A higher percentage of the men (62.2%) had poor HbA1c values as compared to women (46.5%).

For men, 30.1% reported that T2D mostly prevented their activities of daily living as compared to 26.8% reported among the women. Nearly half percentage of the women (53.5%) and men (50.3%) had moderate ability to manage diabetes positively. More women (79.6%) were on prescribed oral hypoglycemic agents as compared to men (70.6%); while the remaining participants were on insulin.

### 4.2. Predictors of QoL and Health State among Men and Women (Tables 2–5)

Women had slightly higher mean QoL scores (*p* < 0.05) for age, schooling, prevention of activities of daily living, ability to manage positively, and knowledge of diabetes and its management as compared to men (Table 2). Women had higher mean health state scores for age and prevention of activities of daily living and knowledge of diabetes and its management as compared to men. Schooling and ability to manage positively were highly significant predictors of QoL among women (*p* < 0.05) as compared to men. Age, prevention of activities of daily living and knowledge/management of diabetes were significant predictors of Health state among women as compared to men. Ability to manage diabetes positively was a significant predictor of health state among men as compared to women.

Schooling and ability to manage diabetes positively were significant with QoL among women; while age, prevention of activities of daily living, and knowledge of diabetes and management were significant with health state among women as compared to men (Table 2). Ability to manage diabetes positively was significant with health state among men compared to women.

Women had higher mean QoL scores for duration of diabetes, diabetes education, and medication as compared to men (Table 3). Positively higher perception on health state scores was found with duration of diabetes, SCA, diabetes education, medication, BMI, and HbA1c among women. SCA and medication were highly significant predictors of health state among women; while BMI was highly significant among men. SCA and medication were significant with health state among women; while BMI was significant with health state among men (Table 3). In this study, quality of life and health state were interdependent variables. MANOVA models were used with all determinants emerging from the ANOVA tests as predictors of QoL and health state (Tables 2–5). These personal and clinical characteristics interact with the specific domains valued as important in life, which explains the significant differences in QoL and health state among Omani men and women.

A further GLM technique was useful to explore the relationship between QoL and health state, interdependent variables with the predictors (like age and duration of diabetes) as seen in Tables 4 and 5. The combined effect of predictors on QoL and health state using Wilks's lambda multivariate tests (Table 4) shows duration of diabetes, prevention of activities of daily living, and ability to manage positively were significant predictors of QoL and health state among men as compared to women.

The test of overall model significance (Table 5) showed the model is important for each dependent variable (QoL and health state). MANOVA models were used with all independent variables in the ANOVA tests as predictors of QoL and health state (Table 5). MANOVA results are explained with the test of overall model significance and the test of overall individual effects of predictors. Among women with T2D, 30.6% of the variance in the total QoL and 14% of the variance in health state could be explained by personal and clinical characteristics (Table 5), while 35.7% of the variance in the total QoL and 23% of the variance in health state was explained by personal and clinical characteristics among men. These *R* ^2^ values indicated a supportive relationship among the predictors of QoL and health state. QoL and health state scores were strongly correlated with the age, diabetes duration, and prior diabetes education. Hence, personal and clinical characteristics had a significant positive effect on QoL and health state supporting the self-care diabetes management model.

“Tests of between-subjects effects” (Table 5) apply an *F* test of significance to the relation of each covariate (age, diabetes duration, SCA, diabetes education, ability to manage positively, and BMI) in relation to each of the dependent variables (QoL and health state). Age and diabetes education were significant predictors of QoL, and SCA was a significant predictor of health state among women as compared to men. Duration of diabetes, diabetes education, ability to manage diabetes positively, and BMI were significant predictors of health state among men as compared to women.

## 5. Discussion

Higher schooling, increased ability to manage diabetes, higher age, moderate level of prevention of activities of daily living, higher knowledge of diabetes and management, higher SCA, and use of medication among women influenced their QoL and health state as compared to men. This state contributed to an increased QoL and health among women as they overcome challenges in coping with T2D. Results of the study were congruent with previous studies [49, 50] that show better self-care leads to improved QoL. In this study, men with T2D had low QoL which was consistent with a previous study [51].

In middle aged women, perceived diabetes did not prevent their activities of living, and they showed above average knowledge and management of diabetes. A strong effect was found for interactions between females and QoL due to higher schooling and ability to manage diabetes positively; while higher age, prevention of ADL, and knowledge/management of diabetes were significant with health state among women. SCA, diabetes education, and medication significantly predicted health state among women. Better physical activity among women contributes to higher QoL and had better understanding of their diabetes. These findings were consistent with other studies [52] that show younger age [53], education [54], longer duration of DM, fasting glucose levels [55], strong knowledge [56], and positive attitude [57–59] had significantly explained higher QoL scores.

Men had consistently lower QoL for all domains compared to women. Poor QoL can prevent men with T2D from achieving improved glucose control. Specific elements like ability to manage positively and BMI influenced QoL and health state among men as compared to women. In turn, low QoL affects HbA1c. Hence, better HbA1c and SCA are major predictors of QoL and health state. Independent predictors can have a contradictory effect on different aspects of QoL. Some studies show that men can experience more restrictions in daily life than women due to unexplained physical and emotional problems [57, 60].

An important finding is an impact of higher ability to manage diabetes positively and prevention of prevents activities of daily living that significantly predicted QoL; while BMI significantly predicted health state among men. The effects were stronger for those with high school and diploma level education and longer duration of diabetes, prevention of activities of daily living, ability to manage positively, and body mass index which were significant determinants among men. Some men have more self-confidence in their ability to manage diabetes and are less likely to be depressed or anxious. Hence, good knowledge and a positive attitude are predictors of adherence to self-care and promote QoL. Men with higher educational levels, strong knowledge, and positive attitude had a higher probability of attaining greater QoL scores [56]. Age [61], psychological perception, SCA, HbA1c, and lower levels of physical activity [62] were significantly associated with higher QoL among men.

The most striking difference was that women had higher QoL scores and health state for higher age and low BMI and adhered better to oral medications and SCA. This finding reflects the inclination of women with higher education to participate in their own self-care. Age, duration of diabetes, diabetes education, and ability to manage diabetes had higher QoL and health state among men. Hence, chronicity of T2D has a differential impact on QoL and health state among women and men. Patients of both genders with lower HbA1c values were shown to have better QoL [63]. Similar studies show high BMI is a strong predictor of decreased QoL [57, 64] and lower BMI was associated with higher QoL [65]. Insulin and higher BMI were associated with lower QoL [33, 61, 63]. QoL and health state scores were lower in women compared with men and lower with longer duration of T2D [54]. HbA1c and QoL have a significant association in previous studies [66, 67]. Higher age, income, and education had better QoL among women [56, 61, 67, 68]. Men and women who have good health have significant health states with T2D [52, 69].

The study limitations are interactive effects of psychological and clinical predictors that may be relevant for comprehensive understanding of the impact on the domains of health-related QoL among men and women.

## 6. Conclusions

The amount of variance influenced by the personal and clinical factors and explained in the GLM is useful in understanding how HRQoL influences Omani men and women. Poor glycemic control increases the risk of developing long term complications of T2D, which causes poor health state and QoL. Maintaining HbA1c within a desirable range is an indicator of good glycemic control and was a contributor to better QoL.

The assessment of QoL and health state is a key component of the self-care management model (Figure 1). This assessment is culturally specific and may assist in early identification to allow for appropriate self-care among individuals with T2D who are at risk for decreased QoL. This study gives useful information to help design appropriate culturally specific interventions related to various aspects of QoL [70]. The SCM model approach indicates that adults need to use their self-care behaviors for goal attainment and to take control of T2D, thus enhancing HRQoL.

This study provides important QoL evidence that may help the diabetes nurse educator (DNE) to identify adults who are at risk of low QoL and develop interventions for healthy lifestyle behaviors based on personal needs, clinical characteristics, and health state. The DNE can educate assist in motivating the patient with T2D to control blood glucose levels, have an annual screening examination, report any changes in health immediately, and engage in rigorous SCM. An effective SCM model empowers men and women in proactively managing T2D and finding ways to overcome the problems with mobility, usual care, self-care, anxiety, and pain. SCM interventions by the DNE should be tailored to the individual taking into account personal needs and motivation to change as well as clinical factors that influence better QoL (Figure 1). The nurse as an educator has an opportunity to positively influence outcomes (QoL and health state) by using effective behavioral skills and a collaborative health care approach.

## Figures and Tables

**Table 1:** Personal and clinical characteristics among Omani men and women (*N* = 300).

Number	Variables	Categories	Men *N* = 143		Women *N* = 157	
			Frequency	Percent	Frequency	Percent
1	Age (years)	30–39	18	12.6	29	18.5
		40–49	39	27.3	64	40.8
		50–59	58	40.6	34	21.7
		60 & above	28	19.6	30	19.1

2	Schooling	Until 8th grade	35	24.5	59	37.6
		High school	59	41.3	58	36.9
		Diploma	48	33.6	38	24.2
		Technical	1	0.7	2	1.3

3	Knowledge of diabetes and its management	Poor	14	9.8	2	1.3
		Fair	11	7.7	14	8.9
		Satisfactory	26	18.2	25	15.9
		Above average	22	15.4	43	27.4
		Good	55	38.5	51	32.5
		Very good	14	9.8	16	10.2
		Excellent	1	0.7	6	3.8

4	Duration of diabetes	0–9	57	39.9	55	35.0
		10–19	67	46.9	77	49.0
		20 & above	19	13.3	25	15.9

5	Self-care activities (SDSCA)	0–3 days/week	88	61.5	95	60.5
		4–7 days/week	55	38.5	62	39.5

6	Diabetes education	No	50	35.0	65	41.4
		Yes	93	65.0	92	58.6

7	Body mass index (kg/m^2^)	<18.5/Underweight	4	2.8	4	2.5
		18.5–24.9/Healthy weight	96	67.1	106	67.5
		25.0–29.9/Overweight	43	30.1	47	29.9

8	HbA1c (%)	<7.0%	54	37.8	84	53.5
		>7.0%	89	62.2	73	46.5

**Table 2:** Personal characteristics and total QoL and health state among men and women.

Determinants	Categories	Men's QoL		Women's QoL		Men's health state		Women's health state	
		Mean	Sig/*p*	Mean	Sig/*p*	Mean	Sig/*p*	Mean	Sig/*p*
Age	30–39	12.22	0.000^*∗*^	12.38	0.000^*∗*^	69.44	0.523	76.38	0.000^*∗*^
	40–49	10.05		10.45		69.36		67.34	
	50–59	8.93		8.50		70.09		73.09	
	60 & above	9.29		8.73		66.61		66.33	

Schooling	Until 8th grade	9.89	0.482	9.85	0.002^*∗*^	67.86	0.425	70.76	0.895
	High school	9.90		11.07		70.68		69.66	
	Diploma	9.35		8.84		68.02		69.47	
	Technical	11.00		10.00		75.00		72.50	

DM prevents activities of daily living	Never	12.17	0.000^*∗*^	10.24	0.000^*∗*^	74.17	0.291	73.33	0.036^*∗*^
	Rarely	11.12		11.60		66.52		65.83	
	Sometimes	10.29		12.29		66.43		71.79	
	Moderately	8.68		9.00		70.36		69.32	
	Mostly	9.00		8.38		68.95		71.07	
	Always	8.75		9.25		70.00		75.00	
	Everyday	0		11.00		0		70.00	

Ability to manage positively	Poor ability	9.22	0.590	10.46	0.058^*∗*^	65.00	0.006^*∗*^	65.77	0.346
	Moderate ability	9.88		9.80		67.01		70.36	
	Good ability	9.61		10.57		72.18		70.89	
	Excellent ability	0		7.00		0		66.25	

Knowledge of DM and its management	Poor	10.86	0.001^*∗*^	8.00	0.003^*∗*^	72.50	0.296	82.50	0.050^*∗*^
	Fair	11.45		11.64		64.09		71.43	
	Satisfactory	9.58		11.16		68.27		65.20	
	Above average	9.73		9.00		68.18		70.47	
	Good	8.96		9.69		69.45		71.47	
	Very good	10.43		10.38		70.36		68.13	
	Excellent	10.00		12.33		85.00		73.33	
	Total	9.72		10.06		69.13		70.06	

^*∗*^ *p* < 0.05 is the level of significance (sig).

**Table 3:** Clinical characteristics and total QOL and health state among men and women.

Variables	Categories	Men QoL		Women QoL		Men health state		Women health state	
		Mean	Sig/*p*	Mean	Sig/*p*	Mean	Sig/*p*	Mean	Sig/*p*
Duration	0–9	8.81	0.000^*∗*^	8.98	0.002^*∗*^	70.70	0.013^*∗*^	70.00	0.001^*∗*^
	10–19	10.15		10.73		69.55		72.14	
	20 & above	10.95		10.36		62.89		63.80	

Self-care activities	0–3 days/week	9.80	0.589	10.02	0.848	69.94	0.226	68.68	0.035^*∗*^
	4–7 days/week	9.60		10.11		67.82		72.18	

Prior diabetes education	No	10.46	0.002^*∗*^	10.58	0.056^*∗*^	66.70	0.036^*∗*^	67.15	0.002^*∗*^
	Yes	9.32		9.68		70.43		72.12	

Medication	OH	10.09	0.002^*∗*^	10.40	0.003^*∗*^	69.55	0.686	70.04	0.003^*∗*^
	Insulin	9.03		8.72		68.44		70.16	
	OH and insulin	8.20		0		67.00		0	

Body mass index (kg/m^2^)	<18.5/Underweight	9.75	0.875	10.00	0.885	66.25	0.007^*∗*^	76.25	0.390
	18.5–24.9/Healthy weight	9.78		9.98		67.45		70.24	
	25.0–29.9/Overweight	9.58		10.23		73.14		69.15	

HbA1c	<7%	9.52	0.371	9.76	0.174	69.54	0.708	70.95	0.241
	>7%	9.84		10.40		68.88		69.04	

^*∗*^ *p* < 0.05 is the level of significance (sig).

**Table 4:** Combined effect of predictors on QoL and health state: multivariate tests/GLM.

Wilks's lambda	Men			Women		
Effect	Value	*F*	Sig./*p*	Value	*F*	Sig./*p*
Intercept	0.586	45.957	0.000^*∗*^	0.607	46.602	0.000^*∗*^
Age	0.965	2.391	0.050^*∗*^	0.804	17.581	0.000^*∗*^
Schooling	0.995	0.307	0.736	0.996	0.300	0.741
Years of diabetes	0.927	5.155	0.007^*∗*^	0.977	1.706	0.185
Self-care activities	0.996	0.252	0.778	0.974	1.950	0.146
Diabetes education program	0.937	4.340	0.015^*∗*^	0.961	2.892	0.050^*∗*^
DM prevents activities of daily living	0.898	7.365	0.001^*∗*^	0.983	1.235	0.294
Ability to manage positively	0.953	3.210	0.044^*∗*^	0.998	0.112	0.894
Knowledge of diabetes/management	0.995	0.306	0.737	1.000	0.034	0.966
Medications	0.981	1.249	0.290	0.993	0.528	0.591
Body mass index	0.926	5.176	0.007^*∗*^	0.968	2.377	0.050^*∗*^
HbA1c	0.993	0.428	0.653	0.988	0.877	0.418

^*∗*^ *p* < 0.05 is the level of significance (sig).

Design: intercept + age + schooling + duration of diabetes + self-care activities + diabetes education program + perceiving DM prevents activities of daily living + ability to manage positively + knowledge of diabetes and its management + medications + BMI + HbA1c.

**Table 5:** Overall model significance and tests of between-subjects effects.

Source	Dependent variable	Men			Women		
	Outcomes	Mean Square	*F*	Sig./*p*	Mean Square	*F*	Sig./*p*
Corrected model	QoL	20.219	6.615	0.000	36.888	5.822	0.000
	Health state	307.235	3.550	0.000	204.893	2.146	0.021

Intercept	QoL	152.364	49.849	0.000	184.703	29.152	0.000
	Health state	3067.884	35.452	0.000	5790.067	60.638	0.000

Age	QoL	0.798	0.261	0.610	181.721	28.681	0.000^*∗*^
	Health state	376.955	4.356	0.039^*∗*^	524.830	5.496	0.020^*∗*^

Schooling	QoL	0.509	0.166	0.684	0.012	0.002	0.966
	Health state	42.830	0.495	0.483	57.694	0.604	0.438

Years of diabetes	QoL	16.577	5.424	0.021^*∗*^	20.945	3.306	0.050^*∗*^
	Health state	501.693	5.798	0.017^*∗*^	18.774	0.197	0.658

Self-care activities	QoL	0.750	0.245	0.621	4.686	0.740	0.391
	Health state	19.141	0.221	0.639	290.368	3.041	0.05^*∗*^

Diabetes education	QoL	5.044	1.650	0.201	18.808	2.968	0.05^*∗*^
	Health state	658.313	7.607	0.007^*∗*^	298.142	3.122	0.050^*∗*^

DM prevents activities of daily living	QoL	43.917	14.369	0.000^*∗*^	9.693	1.530	0.218
	Health state	12.687	0.147	0.702	102.130	1.070	0.303

Ability to manage positively	QoL	1.641	0.537	0.465	0.253	0.040	0.842
	Health state	485.729	5.613	0.019^*∗*^	18.416	0.193	0.661

Knowledge of diabetes and its management	QoL	1.706	0.558	0.456	0.045	0.007	0.933
	Health state	2.853	0.033	0.856	5.747	0.060	0.807

Medications	QoL	7.523	2.461	0.119	0.206	0.033	0.857
	Health state	1.027	0.012	0.913	99.743	1.045	0.308

Body mass index	QoL	0.014	0.005	0.946	15.544	2.453	0.119
	Health state	893.502	10.325	0.002^*∗*^	243.893	2.554	0.112

HbA1c	QoL	2.173	0.711	0.401	10.261	1.620	0.205
	Health state	17.990	0.208	0.649	18.486	0.194	0.661

Generalized linear model: ^*∗*^ *p* < 0.05 is the level of significance (sig). Computed using alpha = 0.05.

Men: QoL *R* ^2^ = 0.357 (adjusted *R* ^2^ = 0.303); health state *R* ^2^ = 0.230 (adjusted *R* ^2^ = 0.165).

Women: QoL *R* ^2^ = 0.306 (adjusted *R* ^2^ = 0.254); health state *R* ^2^ = 0.140 (adjusted *R* ^2^ = 0.075).

## Summary Box

1. What does this paper contribute to the community? Women had higher mean QoL scores for age, schooling, prevention of activities of daily living, ability to manage positively, and knowledge of diabetes and its management.
2. Women had higher mean health state scores for age and perceiving DM prevents activities of daily living and knowledge of diabetes and its management.
3. Age, duration of diabetes, diabetes education, self-care activities, ability to manage diabetes positively, and BMI were significant predictors of QoL and health state among women and men.
